# Knowledge attitude and practice towards infection control measures amongst healthcare workers in a medical teaching hospital of Calicut District, Kerala, India

**DOI:** 10.1186/2047-2994-4-S1-P270

**Published:** 2015-06-16

**Authors:** A Sha

**Affiliations:** 1 General Medicine, Al Iqbal Hospital, Thrissur, India

## Introduction

Healthcare workers must know the various measures for their own protection. They should improve organization of work, implement standard precautions and dispose biomedical waste properly to prevent occupational exposure. This study aimed at assessing the Knowledge and attitude towards Infection control measures amongst the healthcare workers in a medical teaching hospital of Calicut district, Kerala, India.

## Objectives

The objective of the study was to assess the Knowledge and attitude towards Infection control measures amongst the healthcare workers in a medical teaching hospital of Calicut district, Kerala, India.

## Methods

This cross-sectional studywas conducted by using a pretested semi-structured proforma, by interview cum observational technique. One hundred and twenty healthcare workers (70 hospital staff including nurses and technicians and 50 medical interns) were selected using convenient sampling and their Knowledge, attitude and practice towards infection control measures were studied.

## Results

Of the 120 participants, the majority (85.8%) was aware of disposing used needles and syringes in puncture-resistant containers but only 55.7%were actually practicing it. Three-fourths (75.8%) of the participants were aware about not recapping the needles after use but on observation, only 35.4% were practicing this. All healthcare workers were aware about the indication for using masks and gloves while handling patients, while only 77.1% were using them. We also found that only 61.8% washed their hands after attending every patient, 94.3% cleaned the area with a sterile swab before giving injections and only 35.7% of the labs/wards/operation theatres had three colored bags. Few (11.7%) of the workers have already been exposed to infectious blood samples and some (19.2%) are still not immunized against Hepatitis B.

## Conclusion

There is a need for improvement in the Knowledge, attitude and practice of infection control measures among healthcare workers for both self and patient’s protection. They should also get themselves immunized against Hepatitis B and report accidental exposure to infectious samples to the infection control committee.

## Disclosure of interest

None declared.

